# Analysis of plasma microRNA expression profiles revealed different cancer susceptibility in healthy young adult smokers and middle-aged smokers

**DOI:** 10.18632/oncotarget.7866

**Published:** 2016-03-02

**Authors:** Bing Shi, Hongmin Gao, Tianyang Zhang, Qinghua Cui

**Affiliations:** ^1^ Department of Cardiology, Beijing Military General Hospital, Beijing, China; ^2^ Department of Respiratory Medicine, Beijing Military General Hospital No.263 Clinic, Beijing, China; ^3^ Department of Biomedical Informatics, Centre for Noncoding RNA Medicine, School of Basic Medical Sciences, Beijing Key Laboratory of Tumor Systems Biology, Peking University, Beijing, China

**Keywords:** cigarette smoking, plasma, microRNA, expression profile, cancer

## Abstract

Cigarette smoking is a world-wide habit and an important risk factor for cancer. It was known that cigarette smoking can change the expression of circulating microRNAs (miRNAs) in healthy middle-aged adults. However, it remains unclear whether cigarette smoking can change the levels of circulating miRNAs in young healthy smokers and whether there are differences in cancer susceptibility for the two cases. In this study, the miRNA expression profiles of 28 smokers and 12 non-smokers were determined by Agilent human MicroRNA array. We further performed bioinformatics analysis for the differentially expressed miRNAs. The result showed that 35 miRNAs were differentially expressed. Among them, 24 miRNAs were up-regulated and 11 miRNAs were down-regulated in smokers. Functional enrichment analysis showed that the deregulated miRNAs are related to immune system and hormones regulation. Strikingly, the up-regulated miRNAs are mostly associated with hematologic cancers, such as lymphoma, leukemia. As a comparison, the up-regulated plasma miRNAs in middle-aged smokers are mostly associated with solid cancers, such as hepatocellular carcinoma and lung cancer, suggesting that smoking could have different influences on young adults and middle-aged adults. In a conclusion, we identified the circulating miRNAs deregulated by cigarette smoking and revealed that the age-dependent deregulated miRNAs tend to be mainly involved in different types of human cancers.

## INTRODUCTION

MicroRNAs (miRNAs) are short non-coding RNAs that inhibit gene expression at the post-transcriptional level. Recently, increasing evidence indicates that miRNAs are involved in a variety of biological processes such as cell growth, differentiation, and apoptosis [1]. miRNAs are important regulators in health and disease, their deregulation is thus associated with various diseases [2-5]. Moreover, miRNAs are present in the circulation and circulating miRNAs could be biomarkers of various diseases and targets of disease therapy [6]. In recent years, great efforts have been made to identify the miRNAs associated with cigarette smoking [7], a world-wide habit and an important risk factor for cancer. Indeed, some studies had reported that cigarette smoking changed the miRNA expression profile in cancer and other disease [8-11]. In addition, cigarette smoking induces the change of plasma miRNA expression profiles in healthy subjects [12]. However, current studies have not comprehensively addressed the influence of smoking on the plasma miRNAs of healthy subjects. For example, Banerjee et al. investigated only 80 miRNAs. Most of the subjects in Takahashi et al.' study are middle-aged people. The influence of smoking on plasma miRNAs of young healthy subjects remains largely to be explored. Moreover, it still remains unclear what diseases these miRNAs are associated with. Therefore, further studies are needed to ascertain if more novel miRNAs are associated with cigarette smoking and whether differences exist in miRNA associated functions and cancers. And these miRNAs could be helpful for more-effective cancer diagnoses, prevention, and therapies.

In this study, we used microarray to detect the plasma miRNA expression profiles of healthy young male smokers and non-smokers. As a result, we found that the expression levels of 35 miRNAs were significantly changed in the plasma of smokers. Finally, the enriched functions and associated cancers of the deregulated miRNA were analyzed as well.

## RESULTS

### Altered expression of miRNAs in the plasma of cigarette smokers

**Figure 1:**
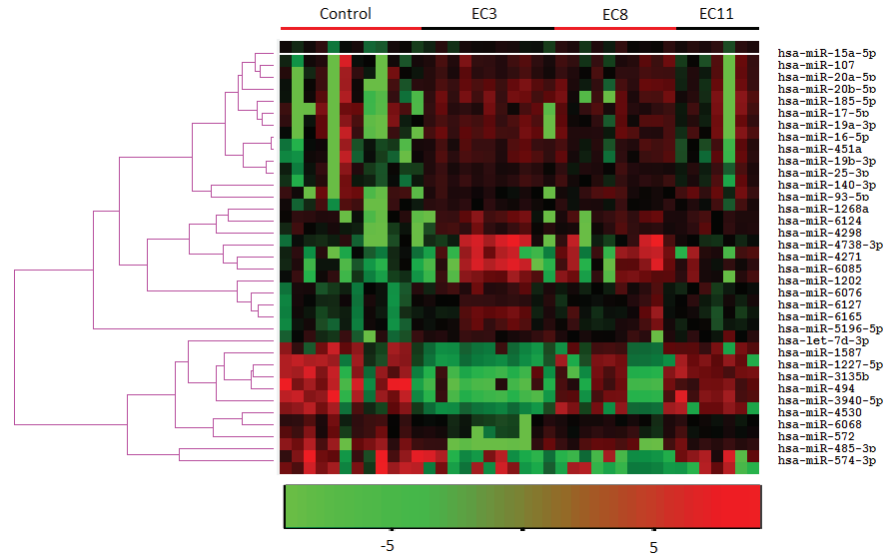
Heatmap of the hierarchical clustering of the 35 differently expressed miRNAs in smokers and non-smokers EC3, EC8, and EC11 represent smokers smoked the cigarette which containing 3, 8, and 11 mg tar, respectively. The hierarchical clustering was performed using the software Cluster 3.0 with default parameters (similarity metric: correlation (uncentered); clustering method: Average linkage).

We determined the plasma miRNA expression profiles in 12 never-smokers and 28 smokers using the Agilent human microRNA array. According to the manufacturer's recommendations, we set up the screening criteria for the differential expression of miRNAs as follows: (a) miRNAs that the average normalized intensities >= 50 in all samples were chosen for differential analysis; (b) the change in expression was more than two fold and the *p*-value (*t* test) was less than 0.05. By this way, 35 differentially expressed miRNAs were selected for the subsequent analyses. A heat map of the hierarchical clustering of the differentially expressed miRNAs is shown in Figure 1. Compared with the never smokers, 24 miRNAs were up-regulated and 11 miRNAs were down-regulated in the smokers (Table 1).

**Table 1:** Deregulated plasma miRNAs in smokers compare to non-smokers

miRNA	Fold	p-value	FDR	deregulation
hsa-miR-107	3.5	0.0408	0.1019	up
hsa-miR-1202	2.3	0.0006	0.0172	up
hsa-miR-1268a	3.1	0.0267	0.0763	up
hsa-miR-140-3p	3.2	0.0210	0.0654	up
hsa-miR-15a-5p	3.0	0.0499	0.1163	up
hsa-miR-16-5p#	3.8	0.0014	0.0192	up
hsa-miR-17-5p#	3.4	0.0470	0.1115	up
hsa-miR-185-5p#	4.2	0.0155	0.0561	up
hsa-miR-19a-3p#	5.4	0.0075	0.0467	up
hsa-miR-19b-3p#	3.0	0.0113	0.0467	up
hsa-miR-20a-5p#	6.0	0.0056	0.0438	up
hsa-miR-20b-5p#	5.0	0.0087	0.0467	up
hsa-miR-25-3p	3.6	0.0027	0.0287	up
hsa-miR-4271	4.8	0.0112	0.0467	up
hsa-miR-4298	5.5	0.0047	0.0391	up
hsa-miR-451a#	3.8	0.0027	0.0287	up
hsa-miR-4738-3p	3.6	0.0326	0.0846	up
hsa-miR-5196-5p	2.1	0.0421	0.1033	up
hsa-miR-6076	2.1	0.0004	0.0153	up
hsa-miR-6085	3.4	0.0191	0.0638	up
hsa-miR-6124	3.6	0.0013	0.0192	up
hsa-miR-6127	2.4	0.0014	0.0192	up
hsa-miR-6165	2.6	0.0001	0.0086	up
hsa-miR-93-5p#	2.6	0.0098	0.0467	up
hsa-let-7d-3p	3.2	0.0111	0.0467	down
hsa-miR-1227-5p	4.2	0.0235	0.0705	down
hsa-miR-1587	4.3	0.0085	0.0467	down
hsa-miR-3135b	5.2	0.0148	0.0561	down
hsa-miR-3940-5p	3.1	0.0326	0.0846	down
hsa-miR-4530	2.2	0.017077424	0.0583	down
hsa-miR-485-3p	5.4	0.0113	0.0467	down
hsa-miR-494	4.9	0.0164	0.0572	down
hsa-miR-572	4.4	0.0207	0.0654	down
hsa-miR-574-3p	8.2	0.0011	0.0192	down
hsa-miR-6068	2.7	0.0273	0.0764	down

### Enriched functions and disease of the deregulated miRNAs

**Figure 2:**
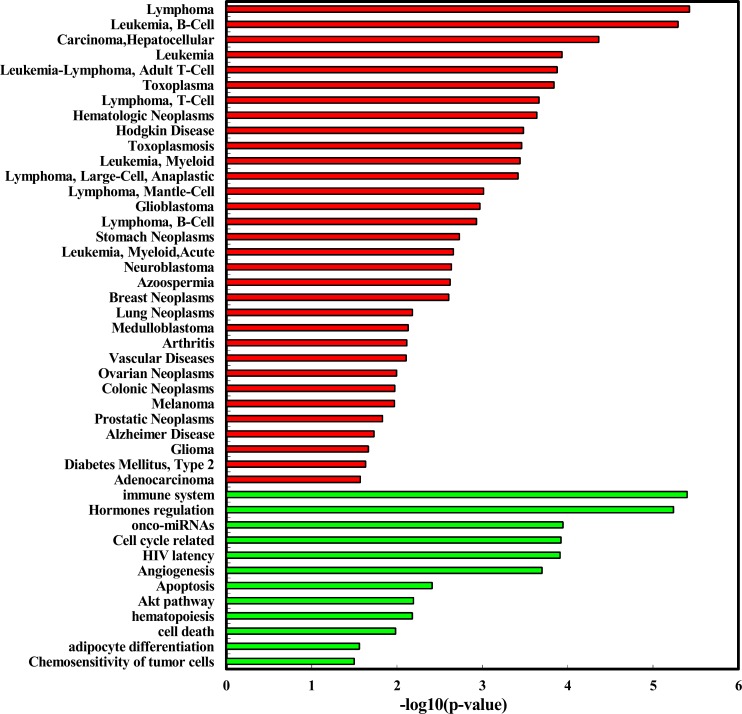
Enriched function (red bar) and diseases (green bar) of the up-regulated miRNAs identified in healthy young adult smokers from this study

**Figure 3:**
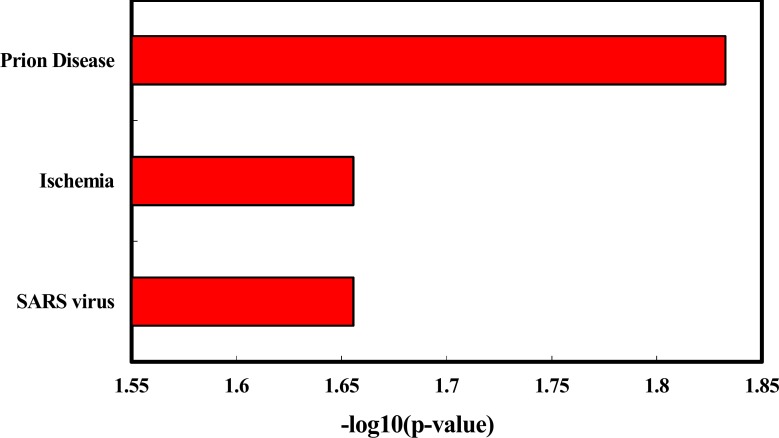
Enriched diseases of the down-regulated miRNAs identified healthy young adult smokers from this study

We explored the enriched functions and associated human diseases of the 35 deregulated miRNAs using the TAM tool (http://www.cuilab.cn/tam). As a result, for the 24 up-regulated miRNAs, they are significantly enriched in a variety of functions, including immune system (*P* = 3.99e-6, Figure 3) and hormones regulation (*P* = 5.77e-6). Strikingly, the smoking induced plasma miRNAs are mostly associated with hematologic cancers (Figure 2). For example, four of the top five diseases mostly associated with the smoking induced plasma miRNAs belong to hematologic cancers, including lymphoma (*P* = 3.75e-6), B-cell leukemia (*P* = 5.12e-6), leukemia (*P* = 1.17e-4), and Adult T-Cell Leukemia-Lymphoma (*P* = 1.33e-4). Interestingly, lung cancer, a well-known smoking related cancer, is not ranked in the top diseases but is still significant. For the 11 down-regulated miRNAs, we did not identify enriched functions but found four significantly associated diseases (Figure 3), including prion disease (*P* = 0.02), ischemia (*P* = 0.03), SARS infection (*P* = 0.03), and cocaine-related disorders (*P* = 0.04). To test the robustness of the deregulated miRNAs in the above functional enrichment analysis, a solution is doing randomization test [13]. Here we took the “lymphoma” (the most enriched disease for up-regulated miRNAs) and the “adenocarcinoma” (the significant but with least significant *p*-value enriched disease for up-regulated miRNAs) as cases for randomization tests. For doing so, we first randomly selected 24 miRNAs (the same number as the up-regulated miRNAs) from the miRNA list of the array. We then matched the 24 random miRNAs with the lymphoma miRNA set and the adenocarcinoma miRNA set, respectively. We then counted the number overlapped miRNAs. We repeated the above process for 10,000 times. In the real case, there are 7 overlaps between the up-regulated miRNAs and the lymphoma miRNAs. For adenocarcinoma, the overlap number is 5. As a result, none of the random overlap numbers is greater than or equal to 7 (*P* < 1.0e-4, Randomization test) for lymphoma. For adenocarcinoma, only 17 random overlap numbers are greater than or equal to 5 (*P* = 1.7e-3). The randomization results are consistent with previous results, suggesting that the deregulated miRNA based enrichment analysis is robust.

Moreover, we further analyzed the deregulated plasma miRNAs from Takahashi et al.'s study, in which the authors identified 43 smoking up-regulated plasma miRNAs and 1 smoking down-regulated plasma miRNAs from middle-aged healthy subjects. Totally, our dataset and Takahashi et al.'s dataset have 9 overlapped deregulated miRNAs (Table 1), which are all up-regulated in both studies. Among the 9 overlapped deregulated miRNAs, miR-17-5p, miR-19a-3p, miR-19b-3p, and miR-20a-5p belong to the miR-17~92 cluster, which is known as oncomiR-1 [14]. The miR-20b-5p is another member of the miR-20a-5p family. In addition, miR-16-5p [15], miR-185-5p [16], miR-451a [17], and miR-93-5p [18] are all oncogenic miRNAs. We next performed TAM analysis to Takahashi et al.'s up-regulated miRNAs. Because there is only 1 down-regulated miRNA, we did not perform TAM analysis for this down-regulated miRNA. Globally, both datasets have highly overlapped (38) TAM terms in enriched functions and diseases (Table 2). There are 6 and 9 specific TAM terms for up-regulated miRNAs from the young smokers and middle-aged smokers, respectively. Moreover, the top significant functions associated with the up-regulated miRNAs for middle-aged smokers include immune system (*P* = 3.52e-9, Figure 5) and hormones regulation (*P* = 4.64e-8), which is consistent with our dataset. However, the diseases mostly associated with the Takahashi et al.'s up-regulated miRNAs are solid cancers but not hematologic cancers (Figure 5). The top 5 mostly associated disease are hepatocellular carcinoma (*P* = 8.11e-11), lung cancer (*P* = 1.62e-10), ovarian cancer (*P* = 7.64e-10), colonic cancer (*P* = 5.05e-9), and stomach cancer (*P* = 7.09e-11). For hematologic cancers, although they are not the most significant ones, they still show significant association with the smoking induced plasma miRNAs (Figure 5). The mostly associated cancers tend to be focused on digestive system cancer and gender related cancer. For example, among the 9 cancers in the top 10 diseases, 5 (55.6%) are digestive system cancers (hepatocellular carcinoma, colonic cancer, stomach cancer, pancreatic cancer, and adenocarcinoma) and 2 are gender related cancers (ovarian cancer and breast cancer). Interestingly, although lung cancer is not the mostly associated diseases with smoking induced plasma miRNA for young adults, it is the No.2 significant disease associated with the smoking-induced plasma miRNAs in healthy middle-aged population (*P* = 1.62e-10). In addition, both datasets connected smoking to cardiovascular disease and metabolic syndrome. Indeed, by literature mining, it was well supported that smoking is associated with various cancers [19], cardiovascular diseases [20], and metabolic syndromes [21].

**Table 2:** Enriched functions and diseases of up-regulated plasma miRNAs in young smokers and middle-aged smokers

	Functions & Diseases
Common (38)	Adenocarcinoma, adipocyte differentiation, Akt pathway, Alzheimer Disease, Angiogenesis, Apoptosis, Breast Neoplasms, Carcinoma, Hepatocellular, Cell cycle related, cell death, Chemosensitivity of tumor cells, Colonic Neoplasms, Diabetes Mellitus, Type 2, Glioblastoma, Glioma, hematopoiesis, HIV latency, Hodgkin Disease, Hormones regulation, immune system, Leukemia, Leukemia, B-Cell, Leukemia, Myeloid, Leukemia, Myeloid, Acute, Leukemia-Lymphoma, Adult T-Cell, Lung Neoplasms, Lymphoma, Lymphoma, B-Cell, Lymphoma, Large-Cell, Anaplastic, Lymphoma, T-Cell, Melanoma, onco-miRNAs, Ovarian Neoplasms, Prostatic Neoplasms, Stomach Neoplasms, Toxoplasma, Toxoplasmosis, Vascular Diseases
Yong smokers only (6)	Arthritis, Azoospermia, Hematologic Neoplasms, Lymphoma, Mantle-Cell, Medulloblastoma, Neuroblastoma
Middle-aged smokers only (26)	Adenoviridae Infections, Aortic Valve Insufficiency, Aortic Valve Stenosis, Carcinoma, Renal Cell, Cell division, Cell proliferation, Colorectal Neoplasms, Eclampsia, Heart Failure, Human embryonic stem cell regulation, Leukemia, Lymphocytic, Chronic, B-cell, Lymphoma, Primary Effusion, Mesothelioma, miRNA tumor supppressors, Pancreatic Neoplasms, Pituitary Neoplasms, Polycythemia Vera, Sarcoma, Kaposi, Schizophrenia

**Figure 4:**
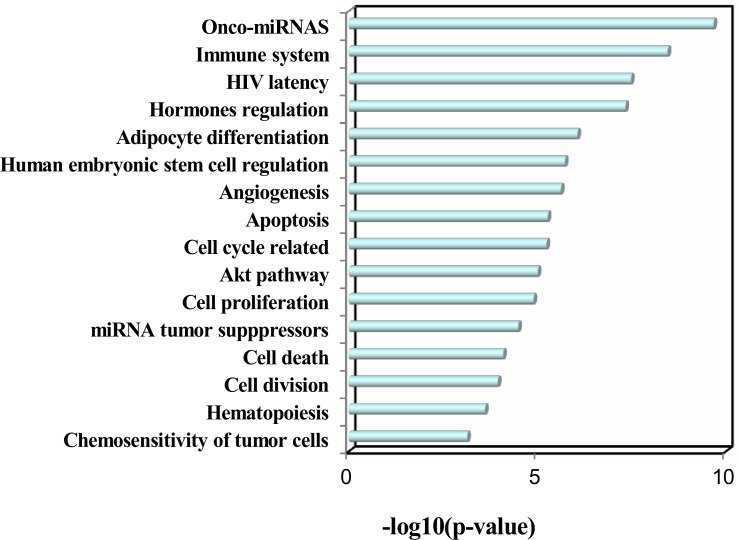
Enriched functions of the up-regulated miRNAs identified in healthy middle-aged smokers from Takahashi et al.' study

**Figure 5:**
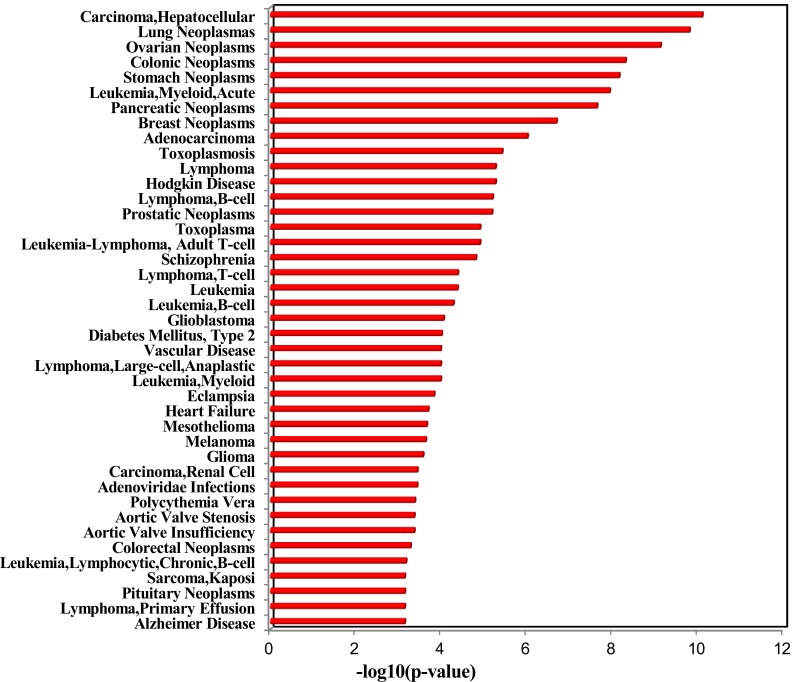
Enriched diseases of the up-regulated miRNAs in healthy middle-aged smokers from Takahashi et al.' study

### Functional and pathway analysis of the targets of the deregulated miRNAs

**Figure 6:**
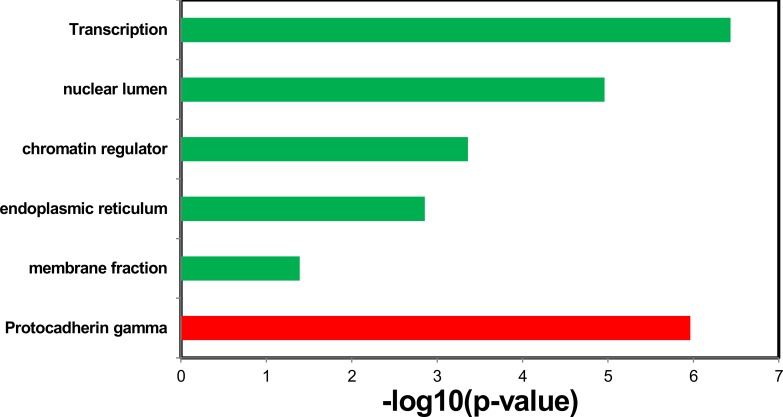
Enriched function of the targets of the up-regulated miRNAs (red bar) and enriched functions of the targets of the down-regulated miRNAs (green bars)

For the up-regulated miRNAs and down-regulated miRNAs, we identified the targets predicted at least by two of the three algorithms, TargetScan, miRanda, and PITA as the candidate targets. We then identified the up-regulated miRNA specific targets and the down-regulated miRNA specific target, respectively. Finally, using DAVID Bioinformatics, we explored the enriched functions of the targets of the deregulated miRNAs. After correcting the p-values by Benjamini, we found the targets of the up-regulated miRNAs are only enriched in the function of Protocadherin gamma (*p*-value = 1.1e-6; Figure 6); whereas the targets of the down-regulated miRNAs are enriched in the functions of membrane fraction (*p*-value = 4.1e-2), endoplasmic reticulum (*p*-value = 1.4e-3), chromatin regulator (*p*-value = 4.4e-4), nuclear lumen (*p*-value = 1.1e-5), and transcription (*p*-value = 3.7e-7, Figure 6).

## DISCUSSION

Tobacco use is a well-established risk factor for cancers and tobacco kills nearly 6 million people each year [22]. Chronic nicotine exposure systemically alters miRNA expression profiles during post-embryonic stages [23], and can lead to the change of miRNA expression profiles in cigarette smokers. In addition, it was reported that the change of miRNA expression profile in adult smokers can affect the miRNA expression of their children and grandchildren [24]. Furthermore, smoking can change plasma microbubbles and miRNA signature [25]. These miRNA changes may contribute to the development of smoking-related cardiovascular pathologies and cancer pathologies. Moreover, these smoking-related miRNA expression changes suggested the possibility to be used as disease-specific biomarkers.

In the present study, 24 up-regulated and 11 down-regulated plasma miRNAs in young healthy smokers were identified by microarray. Moreover, functional enrichment analysis revealed that the up-regulated miRNAs are mostly associated with lymphoma and leukemia, suggesting that lymphoma and leukemia could be two of the most affected diseases by smoking for young adults. Indeed, previous studies have shown that not only smokers are susceptible to leukemia [26], but also the children whose mothers are smokers are also susceptible to leukemia [27]. More interestingly, the smoking induced plasma miRNAs in middle-aged subjects are mostly associated with digestive system cancers and gender related cancers. Although lymphoma and leukemia are also the significant ones affected by smoking for middle-aged population, they are not the most significant ones any more. This suggests that smoking could have different influences for different aged population. Interestingly, previous studies have found that other factors (e.g. calorie restriction) also have age-dependent effects on human health and cancer [28, 29], suggesting that age could be a general factor interacting with various environmental factors and genetic factors.

In a summary, the present study observed that cigarette smoking can change plasma miRNA signature in young adult smokers. Functional analysis revealed that the smoking changed miRNA signature would be risk factors for cancer, cardiovascular disease, and metabolic syndrome. More importantly, smoking could be a serious risk factor especially for leukemia and lymphoma in young adult smokers and especially for digestive system cancer and gender related cancer in middle-aged population. In addition, the uncovered deregulated miRNAs induced by cigarette smoking could be potential biomarkers and targets for smoking-associated diseases.

## MATERIALS AND METHODS

### Subjects

Because most smokers in China are male, all subjects in this study were selected from the male population. 60 smokers aged from 21-30 years old (22.3±3.4yrs) were selected as the cigarette smoking group. The inclusion criteria is as follows: (1) the subjects of cigarette smokers are more than 18 years old; (2) the smoking index is greater than 30 (smoking index = the average number of cigarettes smoked per day × years of smoking history). The exclusion criteria is as follows: (1) currently the smoker is using other tobacco products; (2) history of drug or alcohol abuse; (3) medical history of hypertension, coronary heart disease, diabetes, cerebrovascular disease, pulmonary heart disease, liver disease, kidney disease, respiratory system disease, and blood system diseases. At the same time, 40 male never-smoker aged from 21-30 years old (23.1±4.7yrs) were included as the control group in this study. All subjects participated in the study voluntarily and gave their written informed consent. The study was approved by the Ethics Committee of Beijing military general hospital.

### Plasma sample collection

All subjects are from the same unit and do the same job. All subjects underwent electrocardiogram, blood biochemical examination. Smokers were followed for 56 days after they finished physical examination. During the first two weeks of the study all smokers smoked the same brand of cigarette. During the following 6 weeks, Smokers were divided into three groups (*n* = 20). Smokers from group one smoked the cigarette which containing 3 mg tar (EC3). Smoker from group two smoked the cigarette which containing 8 mg tar (EC8). Smoker from group three smoked the cigarette which containing 11 mg tar (EC11). One half to one pack of cigarettes (the same cigarette brand) were consumed per day by each smoker according to personal habits during the follow-up period. Plasma sample from the smokers were collected in the 56th day of the follow-up period. Plasma sample from the never-smokers were collected after physical examination.

4 mL peripheral venous fasting blood was collected from each subject in EDTA anticoagulant tube. The blood samples were maintained at room temperature for 30 minutes and then centrifuged at 3000g for 10min at 4°C. Then plasma was transferred to another clean EP tube and stored at - 80°C until RNA extraction. Then, the total RNA was extracted from the plasma using the miRNeasy Mini Kit (Qiagen, Hilden, Germany), according to the manufacturer's protocol. The quality and quantity of RNA were measured with a NanoDrop spectrophotometer (NanoDrop Technologies, Wilmington, DE, USA).

### Microarray analysis

In order to investigate the expression profiles of plasma miRNAs among the never smokers and the smokers, plasma miRNA expression profiles were analyzed by CapitalBio (CapitalBio Corp, Peking, China) using the human microRNA array version 19.0 (Agilent, USA). According to the quality of total RNA from plasma, total RNA from 28 smokers and 12 never -smokers were detected with the miRNA array respectively. MiRNAs were labeled using miRNA Complete Labeling and Hyb Kit (Agilent) according to the manufacturer's guidelines. After the labeling procedure was terminated, the Cy3-labeled samples were hybridized according to the instructions for the Agilent microRNA array. Following hybridization, the slides were washed several times and dried according to the manufacturer's guidelines. Then the slides were scanned using the Agilent microarray scanner.

The scanned images were imported into the Agilent Feature Extraction (v10.7) (Agilent Instruments) for grid alignment and data extraction. The replicated miRNAs were averaged and those miRNAs with intensities exceeding 50 in all samples were chosen to calculate the normalization factor. The expression data were normalized using Agilent GeneSpring software. After normalization, the differentially expressed miRNAs between groups were identified with GeneSpring software. The entire datasets (for miRNAs) described here were available from the Gene Expression Omnibus (GEO, http://www.ncbi.nlm.nih.gov/geo/) through series acces sion number GSE69960.

### miRNA functional enrichment analysis

To explore the functions and associated diseases of the deregulated miRNAs induced by cigarette smoking, we carried out miRNA functional enrichment analysis for the deregulated miRNA using the TAM tool [30]. We used the default parameters of TAM. FDR corrections to the P values were used in the analysis.

### Function and pathway analysis of the targets of the deregulated miRNAs

To explore the associated function and involved pathways of the targets regulated by the deregulated miRNAs, we performed functional enrichment analysis to the targets using the DAVID tool. For doing so, we obtained the miRNA-target datasets from TargetScan, miRanda, and PITA. For each deregulated miRNA, only the targets included in at least two of the above datasets are selected for DAVID analysis.
